# Is new tech a pain in the neck? The impact of introducing new technologies in home-care on neck pain: a prospective study

**DOI:** 10.1186/s12889-024-18252-z

**Published:** 2024-03-07

**Authors:** Jan Olav Christensen, Håkon Johannessen

**Affiliations:** National Institute of Occupational Health, Oslo, Norway

**Keywords:** New technology, Homecare, Neck pain, Psychosocial work factors

## Abstract

**Background:**

Home healthcare services are increasingly utilizing novel technologies to enhance quality and efficiency of caregiving, to reduce workloads and compensate for expected labor shortages in the future due to ageing populations. However, rapid, ongoing implementation of new technologies may demand considerable adaptation for employees. The objective of this study was to prospectively examine associations of newly introduced work technologies with neck pain complaints.

**Methods:**

With a nationally representative prospective sample of home-care workers in Norway (N = 887), we estimated effects of 1) introducing new technologies and 2) the appraised quality of training during implementation on neck pain eight months after.

**Results:**

A majority of employees reported new technologies having been introduced the previous 12 months (73.8%). This was not by itself associated with neck pain. However, perceived high quality of training was associated with less subsequent neck pain, also after adjustment for job demands and job control. The strongest effect was seen for “very good” versus “very poor” quality training (OR 0.35, 95% CI 0.17,0.71, in the fully adjusted model). Cross-lagged path analyses ruled out potential reverse causation stemming from the influence of pain on needs for or appraisals of training.

**Conclusion:**

The present findings suggest the introduction of new work technologies has a significant impact on home-care workers’ health, depending on the quality of training during implementation. This highlights the need to include training programs in risk assessments when implementing new technologies.

## Background

Technology is among the most pervasive and noticeable drivers of change in the world of work [1]. “The fourth industrial revolution” has been marked by rapidly increasing levels of digitalization and pervasive use of communication and communication technologies (ICTs). Worries about the *physical* ergonomics of these changes to the ways in which we work have been reflected in terms such as “tech neck” or “technology headaches”, while corresponding concerns pertaining to *psychosocial* risks have given rise to concepts such as “technostress” and “ICT hassles” [2–4]. Hence, during recent decades awareness has been raised of the potentially problematic overuse of electronic devices and the harmful impacts that novel work technologies could have on worker health and well-being [2, 4].

A growing base of research has illuminated the role of non-physical aspects of the work environment for the development, maintenance, and exacerbation of musculoskeletal pain [5]. Drawing in particular on the influential demand-control model of Robert Karasek [6], much of this research has studied psychological job demands and autonomy of workers. While novel work technologies may have unfortunate side effects that cause strain to workers, they also hold a substantial potential to ease and eradicate unhealthy work exposures under the right circumstance, for instance by alleviating job demands while allowing increased worker autonomy and decision authority [7]. However, technologies are usually introduced to improve work processes and production outputs, and evaluating and addressing concomitant effects for workers and work environments may not always be prioritized during implementation.

Home healthcare services have evolved rapidly in recent years, with novel technologies increasingly adopted to improve quality of care as well as efficiency of caregiving [8]. These technologies range from assistive devices for mobility and communication to remote monitoring and telemedicine systems [9]. In Norway and other Nordic countries, home care is a key element of the healthcare system, with a strong emphasis on promoting patient autonomy and independence [10]. Technological innovation is officially considered a key component of strategies to reduce the workload and compensate for expected lack of healthcare labor in the future due to ageing populations [10]. This has led to a greater focus on technologies that support patient self-management and enable remote monitoring, such as mobile health apps and telemedicine systems, and robotic and assistive technologies to support caregivers and help patients with activities of daily living (ADL) [11, 12]. Despite these efforts, there is still a need for further innovation and integration of technologies in the home care sector, particularly in rural and remote areas where access to care can be limited.

For workers, the rapid, ongoing implementation of new technologies may demand considerable learning and adaptation, and disruptions to established ways of working may be perceived as a threat to valued features of the job (i.e. qualitative job insecurity [13]). While worries over novel work technologies have been notable ever since the first industrial revolution [14, 15], recent decades have seen an increase in the attention devoted specifically to information- and communications technologies (ICTs). The role of ICTs in the digitalization of everyday work processes is becoming more salient, and the 2020 pandemic crisis, with the resulting proliferation of remote work, highlighted the role of digital technologies in contemporary work environments.

“Technostress”, denoting psychological challenges associated with interacting with novel work technologies, has been found to be associated with several adverse outcomes, e.g. low organisational commitment, job disssatisfaction, negative affect, burnout and even bullying [7]. In contrast, socalled “technostress inhibitors” have been shown to be associated with positive outcomes such as job satisfaction and organisational commitment [7]. This highlights the dual potential of new technologies, and the potential gains from actively monitoring and controlling possible impacts on workers.

In a longitudinal field study, Elfering et al. [16] found that employee participation in the planning and implementation of an IT project was associated with attenuated risk of back pain six months after, while those with sufficient participation possibilities did not experience any change in back pain. A 2015 study of 46 Norwegian home care workers suggested that the introduction of organisational (job checklists) and technological (personal digital assistants) job aides had no overall effect on musculoskeletal complaints over two years, but that a subgroup of low to moderate strain workers showed improvements [17]. However, overall, despite frequently voiced concerns about the dangers of new technologies, consequences for somatic health remain unclear, as few large scale prospective studies have been conducted.

Using a nationally representative sample of home-care workers in Norway, we empirically examined implications of introducing new work technologies for neck pain complaints. Based on previous literature highlighting the role of facilitation and implementation for the strain outcome of new work technologies, we separately assessed the effects of 1) introducing new technologies and 2) the perceived quality of training during implementation.

## Methods

### Procedure and participants

The present study is an observational study based on data from a project involving a cluster-randomized controlled trial with home care service workers from randomly sampled Norwegian municipalities. A comprehensive description of the overall project and study aims is given in the study protocol [18]. The current analyses are based on questionnaire items that were included for purposes not related to the intervention for which the clusters were randomized, and publications from the project so far have suggested no notable difference between the intervention and control groups with regard to psychosocial working conditions, which were the targets of the intervention [19].

Municipalities were invited to participate if they employed between 20 and 100 care workers (to reduce intracluster variability in the intervention study), and if they had not recently had labor inspections (since the intervention involved labor inspections). Hence, during March and April 2019, 132 municipalities were randomly assigned to one of four trial arms, and then informed about the study by letter and email. All employees from home care services of municipalities that agreed to participate were eligible for participation and invited by email. One hundred and four municipalities initially agreed to participate, but eight subsequently dropped out, leaving 96 municipalities that participated throughout. To enhance statistical power it was subsequently decided to broaden the eligibility criterion of municipalities to be between 101 and 200 home care workers employed. This resulted in 48 additional municipalities that were recruited in June 2019, of which 34 agreed to participate. The total number of participating municipalities was thus extended to 129 out of 180 (71.7

Data were collected from each individual participant using a proprietary web-based questionnaire. The survey could be completed in multiple sessions and accessed with a unique code distributed to each participant in advance. The present analyses utilize data collected once two months prior to the intervention and at 6 months after the intervention, during November 2019, resulting in a follow-up time of approximately eight months.

A total of 6997 workers were invited to participate at both T1 and T2. Of these, 1885 (26.9% of invited) volunteered all relevant information at time 1. Furthermore, 887 (47% of the initial sample) employees provided information about the outcome at T2 and were thus defined as “responders”. Due to the high dropout rate, attrition analyses were conducted by regressing nonresponse at T2 on descriptives at T1 for the initial sample. See Table 1.

**Table 1:** Baseline descriptives of the initial sample (N = 2058) and the final prospective sample after dropout (N = 974)

		Initial	Final	Attrition analysis
		n (%)	n(%)	OR [95% CI]
**Gender**	Male	90 (4.4)	37 (3.8)	Ref
	Female	1968 (95.6)	937 (96.2)	0.77 [0.50,1.18]
**Age**	Mean (SD)	43.7 (11.9)	45 (11.6)	**0.85 [0.79,0.91]** ^∗∗,a^
**Percentage employed**	Mean (SD)	80.2 (21.6)	82.7 (19.8)	**0.99 [0.97,0.99]** ^∗^
**Number of years in current position**	Mean (SD)	9.9 (8.5)	10.7 (8.9)	**0.99 [0.99,0.99]** ^∗^
**Education**	1-9 years	66 (3.2)	30 (3.1)	Ref
	10-12 years	979 (47.6)	449 (46.1)	0.98 [0.59,1.62]
	13-16 years	923 (44.8)	457 (46.9)	0.85 [0.51,1.40]
	$>16$ years	90 (4.4)	38 (3.9)	1.14 [0.60,2.17]
**New technology introduced**	Yes	1516 (73.7)	719 (73.8)	Ref
	No	361 (17.5)	173 (17.8)	0.98 [0.78,1.23]
	Not sure	181 (8.8)	82 (8.4)	1.09 [0.80,1.49]
**Quality of training in new technologies**	Very good	248 (12.1)	117 (12.0)	Ref
	Rather good	606 (29.4)	299 (30.7)	0.88 [0.66,1.17]
	Neither good nor poor/not sure	405 (19.7)	181 (18.6)	1.11 [0.84,1.48]
	Rather poor	165 (8.0)	78 (8.0)	0.95 [0.65,1.39]
	Very poor	92 (4.5)	44 (4.5)	1.10 [0.70,1.72]
**Neck pain**	No pain	785 (38.1)	382 (39.2)	Ref
	Light pain	674 (32.8)	324 (33.3)	1.02 [0.83,1.26]
	Moderate pain	522 (25.4)	239 (24.5)	1.12 [0.90,1.40]
	Severe pain	77 (3.7)	29 (3.0)	1.57 [0.98,2.57]

Note: OR: Odds ratio, 95% CI: 95% confidence interval

$$*p<0.05$$

$$**p<0.01$$

^a^ The effect of age on the odds of dropout is for every ten years

Statistically significant estimates given in **bold**

The study was conducted in accordance with the Declaration of Helsinki and was assessed by the Regional Committees for Medical and Health Research Ethics (REK) (2018/2003/ REK Sør-Øst C). Participants provided informed consent.

### Outcomes - pain complaints

Outcome measures were obtained from a previously published symptom checklist encompassing several health complaints [20]. The intensities of neck pain complaints were assessed by asking “have you been bothered by neck pain the previous 4 weeks?”, with optional answers “not bothered” (1), “a little bothered” (2), “rather bothered” (3), and “severely bothered” (4).

### Exposures - new technologies

The *introduction of new work technologies* was measured with the single item “during the previous 12 months, has your workplace introduced new technologies/digital systems to support work tasks that affect your work situation?”, with response options “Yes”, “No”, and “Not sure”.

*Quality of training with new technologies* was measured with the item “if new technologies have been introduced, have you been given sufficient training in the use of new technologies/digital support systems?”, with response options 1 = “No, very poor”, 2 = “No, fairly poor”, 3 = “Neither good nor bad”, 4 = “Yes, fairly good”, 5 = “Yes, very good”, and 6 = “Not sure/do not know”. In order to maintain a scale that is ordered (for the cross-lagged path analyses) 3 = “Neither good nor bad” and 6 = “Not sure/do not know” were combined into 3 = “Neither good nor bad/not sure”.

### Covariates

In order to account for the potential impact of new work technologies on psychosocial working conditions, the psychosocial work characteristics *job decision control* and *quantitative job demands* were included as potential confounders. These factors are pervasive in research pertaining to the psychosocial work environment, and have been shown to be predictive of neck pain [5]. They were measured with scales from a well established questionnaire instrument for psychosocial work characteristics, The General Nordic Questionnaire for Psychological and Social Factors at Work (QPS_Nordic_) [21]. **Quantitative** demands refer to amount of work and time pressure and was measured as the average of four items (example item: “I have too much to do”). **Job decision control** concerns the delegation of autonomy and opportunities to influence planning and decision-making relevant to one’s job tasks and was measured as the average of five items (example item: “If there are multiple ways of executing work tasks, can you yourself choose which method to use?”). Responses on items for both factors were given on a five-point likert scale, ranging from “1 = very seldom or never” to “5 = very often or always”. Cronbach’s $\alpha$ was 0.84 for job demands and 0.77 for job control.

Dependent variables were also regressed on age, gender, level of education, number of years worked in current position, percentage employment of full time equivalents, and baseline levels of the outcome variable.

### Statistical analyses

Analyses were conducted with R version 4.1.0 [22] and Mplus Version 8.7 [23]. The level of statistical significance was set to $p<0.05$.

First, to establish and quantify potential impacts of 1) the introduction of new technologies and 2) the perceived quality of training, *ordinal logistic regressions* were run with pain complaints at T2 eight months subsequently as outcomes. These regressions were run in two steps, first including age, gender, education, and pain complaints at T1 as covariates, and then adding job demands and decision control as covariates. Regressions were corrected for the potential impact of clustering in municipalities by including random intercepts in the ordinal logistic regressions. This was achieved by running cumulative link mixed models (CLMMs) with the package “Ordinal” in R [24].

**Fig. 1:**
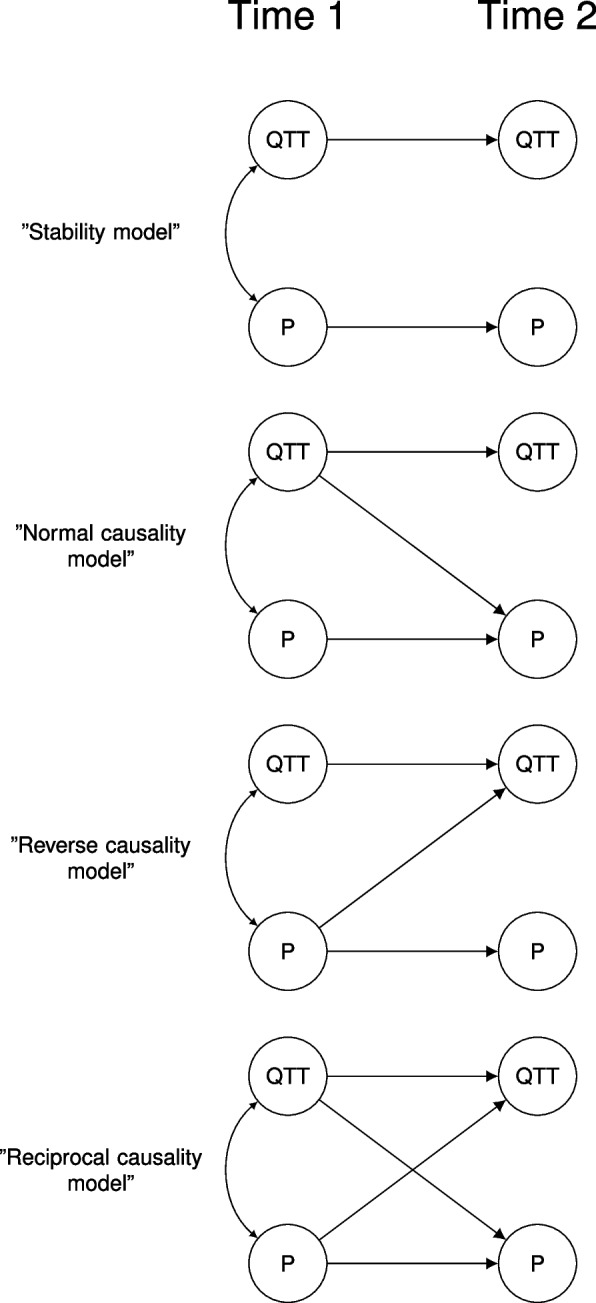
Illustration of the models compared in the cross-lagged path analysis. *Note*: QTT: Quality of technology training, P: Pain complaint

Next, in order to detect or rule out potential reverse causation stemming from the influence of pain complaints on subjective judgments or perceptions of the training for new technologies *cross-lagged path models* were run. Cross-lagged analysis [25] involves modeling bidirectional effects across time. In the current study that implies modeling the effect from T1 quality of technology training to T2 pain complaints (“normal effects”) and from T1 pain complaints to T2 quality of technology training (“reverse effects”). By specifying a series of competing statistical models, comparisons can be made in order to determine which model is the most plausible given the data - i.e. one involving 1) no lagged effects, 2) only “normal effects”, 3) only “reverse effects”, or 4) both normal and reverse effects, also called reciprocal effects (see Fig. 1). Although the existence of causal effects cannot be strictly inferred from observational data with this design, cross-lagged analyses enable a comparison of different causal assumptions, determining which is more likely given the empirical data. Since the more complex models can be specified by adding effect paths to the simpler models, the simpler models are *nested* in the more complex ones, allowing statistical testing of the tenability of adding effect paths. That is, one can statistically determine whether additional effect paths improve model fit to an extent that compensates for the loss of parsimony [25]. An alternative way to consider this analytic strategy is that one starts with the most complicated model, i.e. the reciprocal effects model, in order to judge which paths can be removed from that model without loss of explanatory power. In other words, we reduce complexity until the most parsimonious model that still adequately explains data is reached. Nested models were compared by robust chi-square difference tests.

The cross-lagged models were run in MPLUS, specifying all outcome variables as categorical, invoking a robust weighted least squares estimator (WLSMV), which is appropriate with ordered categorical, non-normally distributed items such as the outcome variables in the current study [23]. Ordinal *probit* regressions were thus employed. Coefficients from probit regressions express the effect of a unit change of the predictor on the z-score of the outcome. The corresponding effect on the *probability* of a level of the outcome is, however, contingent on specific levels of all predictors included in the model. Therefore, probit coefficients are typically not interpreted in terms of magnitude, but only direction and statistical significance.

A sandwich estimator [23] was used to correct standard errors for potential non-independent observations clustered within municipalities.

## Results

Baseline descriptives for the initial and final study samples can be seen in Table 1. Unsurprisingly, the majority of the sample was female (96.2% in the final sample). The average age was 45, and the level of education was centered on upper secondary college (46.1%) and university or college (46.9%) education. The majority of the sample also reported that new technologies had been introduced in their workplace during the previous 12 months (73.8%), with the two largest categories of quality of training being “rather good” (30.7%) and “neither good nor poor/not sure” (18.6%). The majority of the respondents also reported some level of pain (60.8%).

According to our attrition analysis, age, percentage employed, and number of years in current position were statistically significant predictors of dropout (OR for every ten-year increase in age 0.88, 95% CI 0.79,0.91, for percentage employed OR 0.99, 95% CI 0.97,0.99, for number of years in current position OR 0.99, 95% CI 0.99,0.99, Table 1). This suggested the probability of dropping out after baseline was higher for younger workers with less experience and part-time employment. It should also be noted that although the other descriptives did not statistically significantly predict dropout, there were some differences between the initial and the final sample with regard to the higher categories of neck pain (see Table 1).

The ordinal regressions revealed no statistically significant effect of the introduction of new technologies on neck pain eight months after, regardless of whether psychosocial work factors were included (see Table 2). In contrast, for quality of training model 1 exhibited statistically significant effects for all categories compared to “very poor”, with the strongest odds ratio being 0.29 (95% CI: 0.15-0.58) for “very good”. The pattern of associations persisted in model 2, with psychosocial work factors included, expect for the category “not sure” (OR: 0.51, 95% CI: 0.24-1.08).

**Table 2:** Estimates from random intercept ordinal regressions with the introduction of new technologies and the evaluation of implementation as predictors and pain complaints after eight months as outcomes

		Model 1	Model 2
		OR [95% CI]	OR [95% CI]
Introduction of new technologies	None	Ref	Ref
	Not sure	0.87 [0.51, 1.50]	0.82 [0.47, 1.43]
	Yes	0.91 [0.65, 1.28]	0.83 [0.58, 1.19]
Quality of training	Very poor	Ref	Ref
	Rather poor	**0.35 [0.17, 0.70]**	**0.37 [0.18, 0.75]**
	Neither poor nor good	**0.39 [0.21, 0.73]**	**0.44 [0.23, 0.84]**
	Not sure	**0.42 [0.21, 0.86]**	0.51 [0.24, 1.08]
	Rather good	**0.34 [0.18, 0.61]**	**0.39 [0.21, 0.73]**
	Very good	**0.29 [0.15, 0.58]**	**0.33 [0.16, 0.67]**

Note: OR: Odds ratio, 95% CI: 95% confidence interval

**Model 1** was adjusted for the level of outcome at baseline, gender, age, level of education

**Model 2** was adjusted for the level of outcome at baseline, gender, age, level of education, and psychosocial work factors (quantitative job demands and control over decisions)

Statistically significant estimates given in **bold**

The cross-lagged model comparisons suggested the *normal causality* model to be the most tenable: the chi-square difference tests were significant for the normal causality model when compared with the stability model, and for the reciprocal causality model when compared with the reverse causality model, but not compared with the normal causality model (Table 3). This suggests the addition of the normal causality path (the effect of quality of technological training on neck pain) improved the model both when the reversed causality path was present and not, but that the addition of a reverse causality path did not improve the model beyond the basic stability model.

**Table 3:** *P*-values from chi-square difference tests to determine which of the nested path models exhibited the best fit for each pain complaint

		**Stability model**	**Normal causality model**	**Reverse causality model**
	**Model comparison**	$\boldsymbol{\Delta \chi}$	$\boldsymbol{\Delta \chi}$	$\boldsymbol{\Delta \chi}$
		*p*-value	*p*-value	*p*-value
**Neck pain**	Normal causality	**0.01**	-	-
	Reverse causality	0.65	-	-
	Reciprocal causality	-	0.46	$\varvec{<}$**0.01**

Note: Each model is compared to a more complex model. A significant difference test indicates that the more complex model is preferred, i.e. the *additional* paths are justified. *P*-values under the threshold for statistical significance are given in **bold**

Consistent with the chi-square difference tests, the effect estimate for the path from quality of technological training to neck pain was statistically significant, and for the normal causality model this estimate was 0.08 (95% CI: 0.02-0.14, Table 4).

**Table 4:** “Normal” (QTT T1 $\rightarrow$ pain T2) and “reverse” (pain T1 $\rightarrow$ QTT T2) time-lagged effects from path analyses

		Normal causality model	Reverse causality model	Reciprocal causality model
	Path	Est [95% CI]	Est [95% CI]	Est [95% CI]
**Neck pain**	QTT T1 $\rightarrow$ Pain T2	**0.08 [0.02, 0.14]**	-	**0.08 [0.03, 0.14]**
	Pain T1 $\rightarrow$ QTT T2	-	0.02 [-0.06, 0.09]	0.03 [-0.04, 0.10]

Note: QTT: Quality of technology training, Est: Estimate, CI: Confidence interval

Effect estimates are derived from ordinal probit regressions, and only direction and significance should be interpreted. All models are adjusted for age, gender, educational level, number of years employed in current position, and percentage employement. Statistically significant estimates are given in **bold**

## Discussion

The present study aimed to investigate the potential impact of new technology introduction on neck pain among home care services workers, as well as the role of quality of training and psychosocial work factors in this relationship. While no statistically significant effect was observed of the introduction of new technologies as such, a strong effect of the quality of training was observed among those working with novel technologies, and this effect persisted after accounting for job demands and job control. Additionally, we explored the potential causal direction between quality of technological training and neck pain, and these analyses supported the notion of a causal pathway from training to neck pain rather than a reverse causality notion of pain problems affecting the perception of training.

Our findings are in line with previous research that has highlighted the role of organisational resources such as continuous training and involvement facilitation for newly introduced technologies in counteracting technostress and adverse consequences thereof [26]. However, most studies have considered effects on exhaustion, a component of burnout, or mental strain [26], while somatic health outcomes have rarely been studied. Therefore, the present results add to our knowledge about and understanding of the relationship between novel technologies and employee health and well-being. Moreover, the cross-lagged investigation suggested no reverse effects, implying that the association between suboptimal training and neck pain cannot be explained by problems that those who suffer from pain may have in adjusting to new technologies, or their general requirements for more support and facilitation when implementing new ways of working. This adds an important nuance to the findings and highlights the added value of the prospective study design, which has not been common in previous studies relating novel technologies and digitalization to employee health [27].

While the present data do not allow firm conclusions about specific mechanisms, one may surmise that the experience of job control and worker autonomy may be hampered with insufficient training. Job control, i.e. the extent to which the worker feels able to control the resources needed to meet the demands of the job, is one of the most widely studied psychosocial work factors. Low job control has been found in some systematic reviews to predict musculoskeletal disorders, most consistently when combined with high job demands [5]. Increased quantitative workload as a result of having to adapt to new technologies, or technologies creating additional tasks or forcing workers to work faster are also possible and plausible mechanisms [26].

However, in the present study we also observed effects of poor quality training after adjustment for job control and job demands, suggesting other mechanisms are also at play. One could for instance suspect that lack of training has an impact on mechanical ergonomics, so that inadequate training may prevent inappropriate use of new equipment. Moreover, while the quality of training is likely to be important because it prevents misuse and promotes control, it could also be a marker of an organization that cares for its employees, and management that puts emphasis on high quality human resource management. Although the mechanisms are still obscure, human resource primacy has been found to be associated with neck pain in previous studies [28]. In order to further elucidate the why and how of the health-promoting effect of training, future studies should further specify and elucidate the specific training that is being given.

### Methodological considerations

Several strengths of the present study should be noted, such as the prospective design and inclusion of job demands and job control in analyses. However, some limitations are also evident. Perhaps most prominent among these is the general nature of the exposure assessment, i.e. using self-reported single items inquiring on a general level about “new technologies”, without further specification. The consequence of this is that we cannot be sure what type of technology the study pertains to, or what forms of training have or have not been given. Hence, the finding that there was no association of the introduction of new technologies with neck pain cannot be attributed to any specific new technology. Taking this into consideration, finding no association of novel technologies with neck pain is in line with previous research, which has highlighted the diverse nature and consequences of new technologies [27]. For instance, one may expect quite different responses from workers to assistive technologies (presuming they work according to their purpose) and electronic devices for registration and administration of information, and even monitoring of workers. Future research should employ more specific and nuanced measurements to discriminate between various types of work technologies. This is also important with regard to the specific training that should be provided to avoid adverse consequences of new technologies, as there is no general panacea that can be prescribed to all cases. The organization implementing new technology needs to consider the specific characteristics of that technology and the potential and relevant effects it may have on work execution and workers’ perceptions of the work situation.

The response rate was low, and attrition from the initial sample was considerable. If this resulted in a biased sample, it may compromise external validity, i.e. whether the results are valid for the broader population of home care workers. Self-selection bias, where individuals who choose to respond differ systematically from those who do not, can affect external validity. However, selective response can influence results even when response rates are high, while high non-response does not affect validity if it is random [29]. Importantly, we did not have information indicating whether T1 responders were still employed in the same organization at T2, implying that dropout could partly be a result of the high turnover rate in homecare. Our attrition analyses did not suggest any notable difference in the final sample from the initial one. Nevertheless, it is important to acknowledge the limitations of a low response rate and bear this in mind when interpreting results.

All measures were based on self-report. Although the focus of the present study was indeed on the workers’ experience of working conditions, the appraisal of quality of training as well as the judgment of what constitutes “new technology” could vary substantially. Moreover, measuring both exposure and effect by self-report may induce common method bias [30]. The relatively conservative approach, with a prospective design and baseline adjustment for the outcome should attenuate this problem. Nevertheless, future studies should supplement this by using objective measures, which would also enhance the practical utility as it would provide more specific information about technologies and training programs that organizations may consider.

The follow-up period of the present study was eight months, which may be relatively short. As the potential pathogenic mechanisms relevant to the association of new technologies and neck pain are unknown, the possibility remains that effects of novel technologies could have manifested after the second measurement occasion. The statistically non-significant effect that was observed may suggest that if an effect was in fact imminent, it is possible that the technologies in question had risk-attenuating effects and were indeed helpful to the home-care workers. This notion remains speculative, however, and calls for further investigation.

Pain was measured by items asking whether subjects had been “bothered” by pain. The term “bothered” may reflect qualitative aspects of pain that are not reflective of pain intensity, such as the degree to which the individual is affected even by severe pain. However, this phrasing is common in the Norwegian language to convey the intensity of pain. Furthermore, psychometrically, single-item measures are often considered inferior to multiple-item measures, but they do have some advantages. Firstly, they decrease the burden on informants, avoiding perceived redundancy and repetition [31]. Moreover, with multiple items the risk of criterion-contamination increases, i.e. construct-irrelevant information may be included that introduce disturbances rather than relevant variance [31]. Previous research has found single-item verbal pain rating scales to exhibit adequate reliability and validity, e.g. for back pain [32], and in patients with rheumatoid arthritis [33].

### Concluding remarks

Promoting the work ability of home care workers while maintaining high quality care is becoming a pressing concern due to the ageing population. In many cases new technology is used in an effort to streamline work processes and achieve better care. The findings of the present study suggest that the introduction of novel technologies can increase the risk of employee neck pain, but that this is preventable with adequate training. Hence, when facing external pressures to digitalize and implement new technologies, organizations must remain mindful of the importance of adequate, high quality training. This perspective should be included in risk assessments when introducing new technologies, as it may contribute to preventing health-related absences, productivity loss and deterioration of services.

## Data Availability

The datasets generated and/or analysed during the current study are not publicly available due to restrictions defined by the approval given by the Norwegian Centre for Research Data, but will be available from the corresponding author on reasonable request 3 years after study completion. Data access request will be reviewed by NSD - Norwegian Centre for Research Data.
